# Comparative EPR Studies on the Influence of Genistein on Free Radicals in Non-Irradiated and UV-Irradiated MCF7, T47D and MDA-MB-231 Breast Cancer Cells

**DOI:** 10.3390/biomedicines12030518

**Published:** 2024-02-26

**Authors:** Magdalena Jurzak, Paweł Ramos, Barbara Pilawa, Ilona Anna Bednarek

**Affiliations:** 1 Department of Biotechnology and Genetic Engineering, Faculty of Pharmaceutical Sciences in Sosnowiec, Medical University of Silesia, 40-055 Katowice, Poland; ibednarek@sum.edu.pl; 2 Department of Biophysics, Faculty of Pharmaceutical Sciences in Sosnowiec, Medical University of Silesia, 40-055 Katowice, Poland; pawelramos@sum.edu.pl (P.R.); bpilawa@sum.edu.pl (B.P.)

**Keywords:** breast cancer cells (MCF7; T47D; MDA-MB-231), genistein, free radicals, EPR spectroscopy

## Abstract

The antioxidant activity and the association of genistein with carcinogenesis are widely documented. Few studies directly measure the number of free radicals generated in cells, either during the action of factors stimulating their formation, e.g., ultraviolet (UV), or after exposure to antioxidants. The most suitable method for analysing free radicals is electron paramagnetic resonance (EPR) spectroscopy. The EPR method detects a paramagnetic centre with a single electron. Antioxidants neutralize free radicals, therefore, EPR analysis of antioxidant efficacy is as valuable and important as studying the paramagnetic centres of radicals. The aim of the study was to determine the influence of genistein on free radicals basal level and after UV exposure in breast cancer cell lines MCF7, T47D and MDA-MB-231 cell lines. The impact of genistein on cell viability was investigated at concentrations of 0.37 μM, 3.7 μM, 37 μM and 370 μM. Genistein at a concentration of 370 μM revealed a cytotoxic effect on the cells of all three tested breast cancer lines. Genistein at a concentration of 0.37 μM showed no significant effect on the cell viability of all tested breast cancer lines. Therefore, cell proliferation and antioxidant properties were examined using genistein at a concentration of 0.37 μM and 37 μM. X-band (9.3 GHz) EPR spectra of three different types of breast cancer cells (ER-positive, PR-positive and HER-2 negative: MCF7 and T47D and triple-negative MDA-MB-231) were compared. UV irradiation was used as a factor to generate free radicals in cells. The effect of free radical interactions with the antioxidant genistein was tested for non-UV-irradiated (corresponding to the basal level of free radicals in cells) and UV-irradiated cells. The levels of free radicals in the non-irradiated cells studied increased in the following order in breast cancer cells: T47D < MDA-MB-231 < MCF7 and UV-irradiated breast cancer cells: MDA-MB-231 < MCF7 < T47D. UV-irradiation altered free radical levels in all control and genistein-cultured cells tested. UV irradiation caused a slight decrease in the amount of free radicals in MCF7 cells. A strong decrease in the amount of free radicals was observed in UV-irradiated MDA-MB-231 breast cancer cells. The amount of free radicals in T47D cancer cells increased after UV irradiation. Genistein decreased the amount of free radicals in non-irradiated and UV-irradiated MCF7 cells, and only a weak effect of genistein concentrations was reported. Genistein greatly decreased the amount of free radicals in UV-irradiated T47D cancer cells cultured with genistein at a concentration of 3.7 μM. The effect of genistein was negligible in the other samples. Genistein at a concentration of 3.7 μM decreased the amount of free radicals in non-irradiated MDA-MB-231 cancer cells, but genistein at a concentration of 37 μM did not change the amount of free radicals in these cells. An increase in the amount of free radicals in UV-irradiated MDA-MB-231 cancer cells was observed with increasing genistein concentration. The antioxidant efficacy of genistein as a potential plant-derived agent supporting the treatment of various cancers may be determined by differences in signalling pathways that are characteristic of breast cancer cell line subtypes and differences in activation of oxidative stress response pathways.

## 1. Introduction

Epidemiological data show that breast cancer is the second most common type of malignant tumour (after lung cancer). Breast cancer is the most common cancer in women and one of the leading causes of death [1]. Oestrogens, a known risk factor for breast cancer, affect multiple signalling pathways associated with cell proliferation, survival and apoptosis and, therefore, they play a key role in the carcinogenesis of mammary tissue [2]. Oestrogens interact with two subtypes of oestrogen receptors (ERs): ERα and ERβ [3,4]. In addition, most breast cancers in women express ERs and are sensitive to treatment. ER-negative breast cancers are a group of tumours with poor prognosis and lack effective therapeutic strategies compared to ER-positive tumours [5].

**Figure 1:**
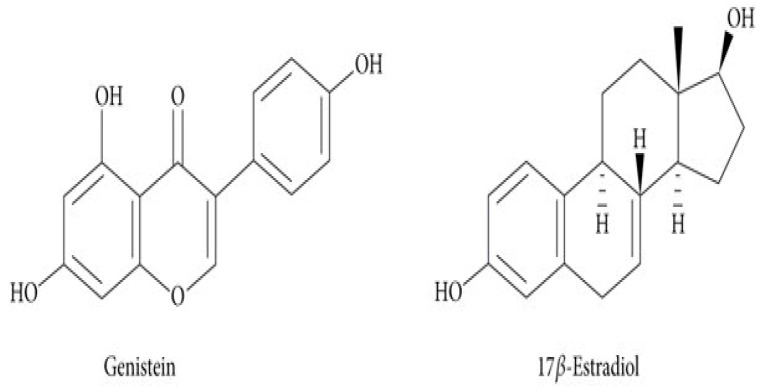
Chemical structure of genistein and 17β-estradiol [13].

The phytoestrogen genistein (4′,5,7-trihydroxyisoflavone), a naturally occurring soybean isoflavone, has a heterocyclic diphenolic structure similar to oestrogens, and an affinity with oestrogen receptors (Figure 1) [6]. Genistein can bind to the ER, but the effects it induces (oestrogenic or anti-oestrogenic) in cells remain unclear [7,8]. However, the chemopreventive and anticancer activities of genistein are well documented and include suppression of tumour cell proliferation through inhibition of protein tyrosine kinase, reduction of oxidative stress-inducing protein, upregulation of adhesion molecules, inhibition of angiogenesis or cell migration, downregulation of telomerase activity and DNA topoisomerase, induction of the expression of cell cycle inhibitors and induction of cell cycle arrest and apoptotic cell death [9].

In contrast, previous studies have shown that genistein has antioxidant properties and is able to prevent apoptotic cell death. Genistein inhibited UV irradiation-induced oxidative stress resulting from free radical production [10].

Free radicals can alter gene expression and directly affect cell proliferation and apoptosis through the activation of transcription factors. Oxidative stress caused by free radicals may participate in all stages of carcinogenesis, from initiation through promotion to progression [11]. In addition, cancer cells have increased levels of free radicals compared to normal cells due to their accelerated metabolism. The high level of free radicals in cancer cells could be protumorigenic, but it is also their vulnerability. The high level of free radicals in cancer cells makes them more susceptible to oxidative stress-induced cell death and can be exploited for selective cancer therapy [12].

It is important to emphasize that the anticancer activity of phytochemicals is cell specific. A phytochemical may be effective in one or more cell lines, and this may be due to differences in the cell component system.

Currently, many breast cancer cell lines are commercially available, varying in molecular features and morphological characteristics [14]. One of the features of breast cancer tumour cells is the presence of three important receptors: oestrogen receptor (ER), progesterone receptor (PR) and human epidermal growth factor receptor 2 (HER2). MCF7 and T47D are HER2-negative, ER-positive and PR-positive breast cancer cell lines [14]. However, the T47D cell line was markedly susceptible to progesterone whereas the MCF7 cell line did not respond to progesterone in the presence of oestrogen [15]. There are also bioenergetic differences between MCF7 and T47D breast cancer cells [16]. The MDA-MB-231 is a triple-negative breast cancer cell line (HER2-negative, ER-negative, PR-negative) [14]. Cell signals mediated by the oestrogen receptors: extracellular signal-regulated kinase (ERK1/2), protein kinase B (Akt/PKB) and nuclear factor kappa B (NFκB); and by human epidermal growth factor type-2 receptors: mitogen-activated protein kinase (MAPK) cascade signalling, phosphatidylinositol polyphosphate (PIP), are connected with the progression and development of breast cancer [17]. All tested breast cancer cell lines (MCF7, T47D and MDA-MB-231) are breast invasive carcinoma; however, cells of these lines show discrepancies between activities of cancer-related pathways. The cells of triple-negative breast cancer MDA-MB-231 revealed significant upregulation of JAK/STAT and MAPK signal transduction pathway. ER-positive MCF7 and T47D breast cancer cell lines, in opposition to MDA-MB-231, demonstrates downregulation of JAK/STAT signal transduction pathway and, additionally, NFkappaB and TNFα mediated signal transduction pathway. Oestrogen-related signal transduction pathways in MCF7 and T47D are upregulated. MAPK signal transduction pathway is downregulated in T47D breast cancer cell line [18].

Today’s diagnostic and therapeutic solutions require a comprehensive understanding of the detailed mechanisms that underlie the disease. The molecular pathomechanism of a disease is crucial not only for the design of appropriate trials but also for the development of effective treatment strategies, which can be achieved through the identification of molecular markers or precise linkage to signalling pathways. Currently, we are investigating the use of molecularly targeted drugs and researching the refined molecular effects of selected compounds/substances. In view of the foregoing, and understanding the importance of molecular cellular differentiation, the study presented here has sought to clarify the response of genistein against three different cell lines representing breast cancer, each of which has its own “unique” molecular substrate associated with the mechanism of tumorigenesis.

The aim of the study was to determine the influence of genistein on viability, proliferation and free radicals basal level, and after UV exposure in breast cancer cell lines MCF7, T47D and MDA-MB-231. 

Intra-cellular basal concentration of free radicals in breast cancer cell lines, antioxidant capacity of genistein to free radicals neutralizing and change in the amount of free radicals after UV-irradiation were measured directly using the modern electron paramagnetic resonance (EPR) spectroscopy. The absorption of microwaves by free radicals in the cancer cells was compared.

The effect of genistein concentration on free radicals in the tested cells cultured with this phytoestrogen was determined. In addition, the changes in free radicals in the cells tested after UV irradiation were examined. The modern electron paramagnetic resonance (EPR) spectroscopy analysis was carried out. The absorption of microwaves by free radicals in the cancer cells was compared.

## 2. Materials and Methods

### 2.1. Cell Lines

Three breast cancer cell lines were used: ER-negative MDA-MB-231, ER-positive MCF7 and ER-positive T47D. MDA-MB-231 metastatic human breast adenocarcinoma cells exhibit a high invasive capacity compared to MCF7 and T47D breast cancer cells [19,20], and MCF7 invasive ductal breast carcinoma cells exhibit high proliferative activity in comparison with T47D metastatic ductal breast carcinoma cells [21].

Human breast adenocarcinoma cell lines: MDA-MB-231 (ATCC^®^ HTB-26™), MCF7 (ATCC^®^ HTB-22™) and ductal carcinoma cell line T47D (ATCC Number^®^HTB-133™) were cultured in Dulbecco’s Modified Eagle’s Medium (DMEM) supplemented with 4 mM L-glutamine, 4500 mg/L glucose, 1 mM sodium pyruvate and 1500 mg/L sodium bicarbonate (ATCC American Type Culture Collection, Manassas, VA, USA) supplemented with 10% foetal bovine serum (FBS, American Type Culture Collection, Manassas, VA, USA) and 1% pen/strep solution (100 U/mL penicillin, 200 µg/mL streptomycin) (Sigma Aldrich, St. Louis, MO, USA). Cells were grown in monolayers to confluence in Nunc Easy Flasks with filter 25 cm2 culture surfaces (T25, Thermo Scientific Nunc, Roskilde, Denmark). After reaching confluence, the cells were passaged with a trypsin 0.25% EDTA solution (1×) (Sigma Aldrich; St. Louis, MO, USA). Cell cultures were maintained at 37 °C in a humidified incubator (95%) with 5% CO_2_.

### 2.2. Genistein

Genistein from Glycine max (soybean) used in the study was purchased from Sigma Aldrich^®^ (cat. no. G6776) and dissolved in dimethyl sulfoxide (DMSO; Sigma Aldrich; St. Louis, MO, USA) so that the final concentration in the experimental wells did not exceed 0.5% (*v*/*v*). An aliquot of a 37,000 µM stock solution of genistein was stored in the dark at −70 °C, thawed and diluted with cell culture medium to the appropriate concentration before use.

### 2.3. Cell Viability Measurement

The cytotoxic effect of genistein was determined by WST-1 test (Roche Diagnostics GmbH, Mannheim, Germany), according to the manufacturer’s protocol. Briefly, MCF7, MDA-MB-231 and T47D breast cancer cells were seeded in 96-well plates at a density of 5 × 10^4^ cells/well. Plates were incubated at 37 °C in 5%CO_2_ for 24 h to allow attachment to the bottom of the wells. After 24 h, the medium was changed to medium supplemented with genistein at the following concentrations: 0 µM (control), 0.37 µM, 3.7 µM, 37 µM and 370 µM.

After 72 h exposure at 37 °C in 5% CO_2_, the medium containing genistein from each well was replaced with fresh medium (100 µL) and 10 µL of WST-1 test (Roche Diagnostics GmbH; Mannheim, Germany) was added to each well. During the 45 min incubation period, any viable cells converted the stable tetrazolium salt (WST-1) into a water-soluble formazan dye. The absorbance value of the formazan product is directly related to the number of metabolically active cells in the cell culture.

After 45 min of incubation, the absorbance of the water-soluble formazan dye in each well was measured using a microplate reader UMV340 (Biogenet Asys Hitech GmbH, Eugendorf, Austria) at 450 nm. All concentrations of genistein were tested in triplicate and the assay was carried out in three separate experiments.

Based on the statistical analysis of the results of the cytotoxicity assay on the cells of the three breast cancer lines (tested concentrations: 370 µM, 37 µM, 3.7 µM, 0.37 µM), two concentrations of genistein were finally used to evaluate the effect of genistein on breast cancer cell proliferation (MDA-MB-231, MCF7 and T47D) and its antioxidant properties: 3.7 µM and 37 µM.

### 2.4. Cell Proliferation Measurement

The CyQUANT^®^ NF (Invitrogen, Thermo Fisher Scientific Inc., Waltham, MA, USA) assay is based on the measurement of cellular DNA content via fluorescent dye binding. As cellular DNA content is highly regulated, it is closely proportional to cell number. The extent of proliferation is determined by comparing the cell counts of samples treated with drugs or other compounds of interest with untreated controls.

For CyQuant proliferation assays, MCF7, MDA-MB-231 and T47D breast cancer cells were seeded in 96-well black plates (Thermo Scientific Nunc, Roskilde, Denmark) at a density of 5 × 10^4^ cells/well. The plates were incubated at 37 °C in 5% CO_2_ for 24 h to allow attachment to the bottom of the wells. After 24 h, the medium was changed to medium supplemented with genistein at the following concentrations: 0 µM (control), 3.7 µM and 37 µM.

After 72 h exposure at 37 °C in 5% CO_2_, the medium containing genistein was gently aspirated from each well. Then 100 µL of 1× dye binding solution was added to each microplate well. The microplate was covered and incubated at 37 °C for 60 min. The fluorescence intensity of each sample was measured using a fluorescence microplate reader (Spectrophotometer Spark 10M, TECAN, Männedorf, Switzerland) with excitation at 485 nm and emission detection at 530 nm. The experiment was performed three times in triplicate.

### 2.5. UV Irradiation

MCF7, MDA-MB-231 and T47D breast cancer cells were seeded in 6-well plates at the density of 1 × 10^5^ cells/well and incubated in standard DMEM medium supplemented with FBS and antibiotic solution. The plates were incubated at 37 °C in 5% CO_2_ for 24 h to allow attachment to the bottom of the wells. After 24 h, the medium was changed to medium supplemented with genistein at the concentrations: 0 µM (control), 3.7 µM and 37 µM.

After 24 h of incubation with genistein, the cells were treated with UV irradiation. The cells were then treated with trypsin 0.25% EDTA, neutralized DMEM with 10% FBS and centrifuged at 1000× *g* for 5 min. The supernatants were discarded. The cell pellets were resuspended in fresh DMEM without FBS and without antibiotics.

### 2.6. EPR Measurements

Free radicals in the cells located in thin-walled glass tubes were examined by an X-band (9.3 GHz) electron paramagnetic resonance (EPR) spectrometer with magnetic modulation of 100 kHz produced by Radiopan Firm (Poznań, Poland). The first-derivative EPR spectra of the cells were acquired numerically by the Rapid Scan Unit of Jagmar Firm (Kraków, Poland). The measurement time of each EPR line was 1 s. The accumulation of the EPR signals of a single sample was performed. MCM101 recorder of EPRAD Firm (Poznań, Poland) measured microwave frequency with accuracy [±0.0002 GHz]. The measurements were performed with attenuation of microwave power of 5 dB, to obtain high microwave power. The total microwave power generated by the klystron—the source of microwaves in the EPR spectrometer—was 70 mW. The EPR study used professional spectroscopy software LabVIEW 8.5.1 (National Instruments Corporation, Austin, TX, USA).

The g-Factors and amplitudes (A) of the EPR spectra were analysed. The amplitude (A) [±0.1 a.u.] depended on the amount of free radicals in the sample [22,23]. The amplitude (A) increased with increasing free radicals. Amplitudes (A) were divided by the volume of the cells in the glass tubes in order to compare the relative amounts of free radicals in the individual cells. The g-values [±0.0002] were determined according to the following formula [22,23]:

g = hν/μ_B_B_r_ (1)

where: h—Planck constant; ν—microwave frequency; μ_B_—Bohr magneton; B_r_—induction of resonance magnetic field. This formula was obtained from the paramagnetic resonance condition. The g-values characterized the type of paramagnetic centres [22,23]. The errors of the EPR parameters were determined by the total differential method, which takes into account the errors of all the measured spectral values.

### 2.7. Statistical Analysis

One-way ANOVA followed by post hoc Dunnett’s test was used to determine significant differences between the mean of viability/proliferation control cells (cells not treated with genistein) and genistein treated cells. The Shapiro–Wilk test for data normality was used. Statistical analysis of the data showed their normality; therefore, the viability (absorbance λ = 450 nm) and the proliferation (fluorescence λ = 530 nm) results are represented as the average (X) ± standard deviation (SD). Values of *p* < 0.05 were considered statistically significant. Statistical analysis was carried out using TIBCO Statistica^®^ 13.6.0 software.

## 3. Results and Discussion

### Cells Viability and Proliferation

**Figure 2:**
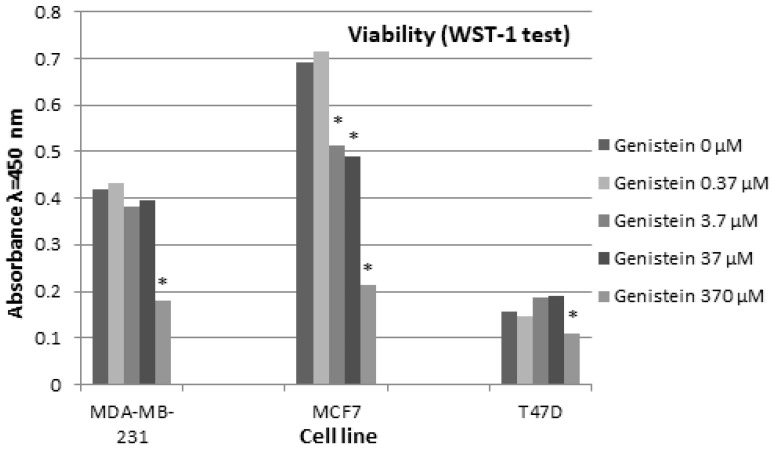
Mean absorbance of MDA-MB-231, MCF7 and T47D cells treated with 0.37 µM, 3.7 µM, 37 µM and 370 µM genistein for 72 h compared to control MDA-MB-231 (genistein 0 µM), MCF7 (genistein 0 µM) and T47D (genistein 0 µM) cells. * *p* < 0.05, post hoc Dunnett’s test.

In this experimental model, the effect of genistein at several concentrations (370 µM, 37 µM, 3.7 µM, 0.37 µM) on breast cancer cell viability was compared to cells not treated with genistein (0 μM), for cells of each breast cancer cell line (MDA-MB-231 0 μM, MCF7 0 μM and T47D 0 μM) (Figure 2).

Genistein at concentration of 370 μM revealed a cytotoxic effect on cells of all three breast cancer cell lines: MDA-MB-231 (0.179 ± 0.01), T47D (0.111 ± 0.017) and MCF7 (0.213 ± 0.014) (*p* < 0.05, post hoc Dunnett’s test).

MDA-MB-231 viability showed no significant differences between control MDA-MB-231 cells (0.418 ± 0.014) and MDA-MB-231 cells treated with 0.37 μM (0.431 ± 0.007), 3.7 (0.383 ± 0.008) and 37 µM genistein (0.396 ± 0.011) (*p* > 0.05, post hoc Dunnett’s test). Similarly, the viability of T47D cells showed no significant differences between control T47D cells (0.156 ± 0.009) and T47D cells treated with 0.37 μM (0.148 ± 0.008), 3.7 (0.186 ± 0.01) and 37 µM genistein (0.189 ± 0.004) (*p* > 0.05, post hoc Dunnett’s test). The viability of MCF7 cells treated with 0.37 μM (0.713 ± 0.004) showed no significant differences in comparison to control MCF7 cells (MCF7 0 μM) (*p* > 0.05, post hoc Dunnett’s test). The viability of MCF7 cells treated with 3.7 µM (0.514 ± 0.011) and 37 µM genistein (0.488 ± 0.009) showed significant differences compared to control MCF7 cells (0.690 ± 0.006) (*p* = 0.0235 and *p* = 0.0178; post hoc Dunnett’s test) (Figure 2).

**Figure 3:**
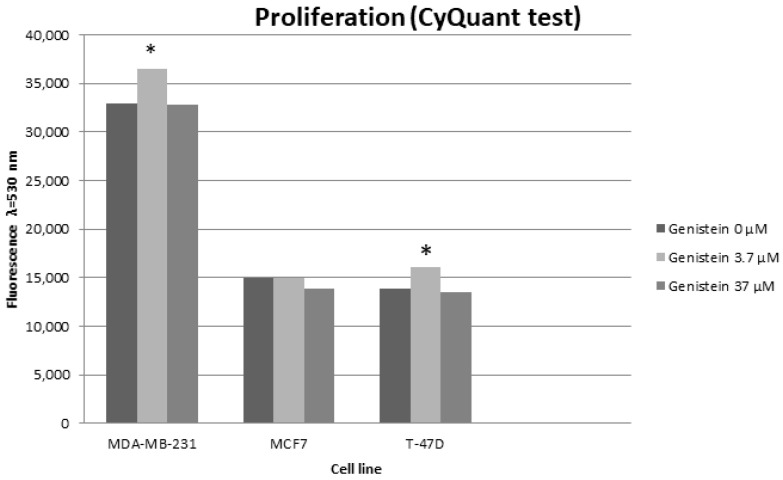
Average fluorescence of MDA-MB-231, MCF7 and T47D cells treated with 3.7 µM and 37 µM genistein for 72 h compared to their respective controls. * *p* < 0.05, post hoc Dunnett’s test.

To examine the proliferation and antioxidant features of genistein, the cells were exposed to concentrations of 3.7 µM and 37 µM. This is because genistein did not affect cell viability at a concentration of 0.37 µM, but had a cytotoxic effect on the tested cells of three breast cancer cell lines at a concentration of 370 µM. The impact of genistein on breast cancer cell proliferation was evaluated by comparing the effects of concentrations of 3.7 μM and 37 μM to untreated cells (0 μM) for each cell line (MDA-MB-231, MCF7, and T47D) (Figure 3).

The proliferation of MDA-MB-231 and T47D cells treated with 3.7 µM genistein (36,552 ± 1106, 16,077 ± 275) exhibited significant differences as compared with control MDA-MB-231 and T47D cells (32,905 ± 989; 13,882 ± 415) (*p* = 0.01523 and *p* = 0.01238, respectively; post hoc Dunnett’s test). The proliferation of MDA-MB-231 and T47D cells treated with 37 µM genistein (32,870 ± 672; 13,449 ± 511) showed no significant differences compared to control cells (*p* > 0.05, post hoc Dunnett’s test). The proliferation of MCF7 cells showed no significant differences between control MCF7 cells (14,934 ± 795) and MCF7 cells treated with 3.7 µM (14,992 ± 1108) and 37 µM genistein (13,869 ± 644) (*p* > 0.05, post hoc Dunnett’s test) (Figure 3).

The response of breast cancer cells to genistein varies widely between cell lines. In vitro and in vivo studies indicate that the dose of genistein and the oestrogen dependency of the cancer cells appear to be the most important factors associated with the SERM (selective oestrogen receptor modulator) property of genistein [24].

Our data indicate a decrease in viability and no effect on proliferation of MCF7 cells treated with 3.7 and 37 µM genistein. Chen et al. [25] reported a significant inhibition of MCF7 cell growth in the G2/M phase and decrease in the proliferative S phase of MCF7 cells treated with genistein at concentrations above 50 µM.

Exposure of MDA-MB-231 and T47D cells to genistein at any concentration applied had no effect on cell viability; however, 3.7 µM genistein concentration increased MDA-MB-231 and T47D cell proliferation. Jordan et al. [26] showed that genistein at 2 µM stimulated cell proliferation in MDA-MB-231 cells, but not at 7 or 12 µM. Similarly, genistein at low concentrations of 5 and 10 µM induced cell proliferation, whereas a high concentration of 50 µM induced cell death [27,28], which seems to confirm our study.

In contrast, Yang et al. [24] showed that genistein inhibited cell growth in MDA-MB-231 Erα-negative/oestrogen-independent cells at both low and high concentrations. In ERα-positive/oestrogen-dependent cells (MCF7, T47D), genistein promoted cell growth at a lower concentration (below 5 μM) while inhibiting growth at a higher concentration (above 10 μM).

Furthermore, Sotoca et al. showed that the cell proliferative effect of genistein is inversely proportional to the ratio of ERα/ERβ in breast cancer cells [9]. According to Choi, ERα expression is downregulated by genistein [29].

On the basis of the available literature data, it is possible to attempt an elucidation of the molecular mechanism of the effect of genistein on individual cell lines, taking into account the effects on proliferation and apoptosis. Numerous signalling pathways converge to modulate the activity of various protein kinases. By affecting an effector protein, the appropriate response of the cell can be observed in terms of “decision making”, resulting in either cell proliferation or its slowdown. In the ER-positive MCF7 cell line, genistein at low concentrations (1 nm to 10 µM) stimulates cell proliferation [25].

However, genistein at concentrations above 25 μM induces apoptosis by increasing the expression of CDKN1A and p53 and decreasing the expression of TNFR, GADD45A, NF-κB, Bcl-2 [30]. In MDA-MB-231 cells proliferation was reduced when genistein was used at concentrations above 20 µM [31,32]. In this case, the activation of apoptosis was mainly due to the downregulating of cyclin B1 and Bcl-2 expression and cessation of NF-κB signalling by the Notch-1 pathway, at 5–20 µM [31], whereas at 5–50 µM, this phytochemical induced apoptosis through the production of reactive oxygen species (ROS) as a result of the reduction of copper ions from Cu(II) to Cu(I) [32]. In our study, we also found an increase in free radicals when the MDA-MB-231 cells were treated with genistein at higher concentrations, as will be shown in the results of the study presented in the following sections. In ERα-positive/oestrogen-dependent cells (MCF7, T47D), genistein promotes cell growth at a lower concentration (below 5 μM), while inhibiting growth at a higher concentration (above 10 μM) [24]. Moreover, Sotoca et al. showed that the cell proliferative effect of genistein is inversely proportional to the ratio of ERα/ERβ in breast cancer cells [9].

Free radicals were found in all cells examined. The changes in the amplitudes (A) reflect the changes in the levels of free radicals in the cells after the interaction with genistein and the exposure to ultraviolet radiation.

**Figure 4:**
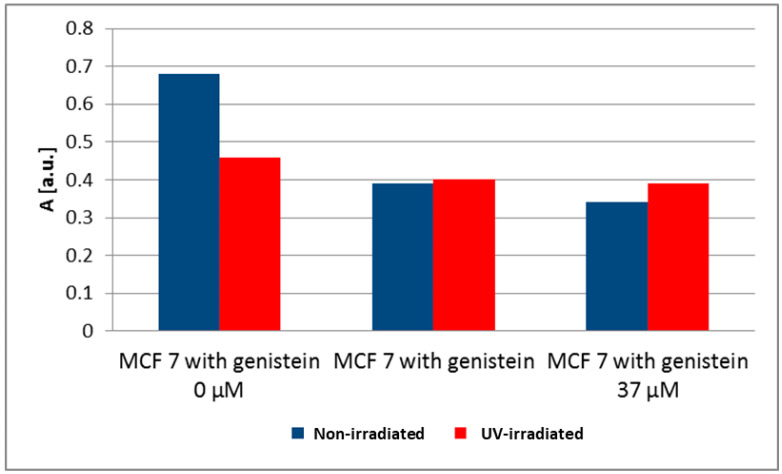
The effect of genistein on the amplitudes (A) [±0.1 a.u.] of the EPR spectra of non-irradiated and UV-irradiated MCF 7 cells. The genistein concentrations were 3.7 μM and 37 μM.

**Figure 5:**
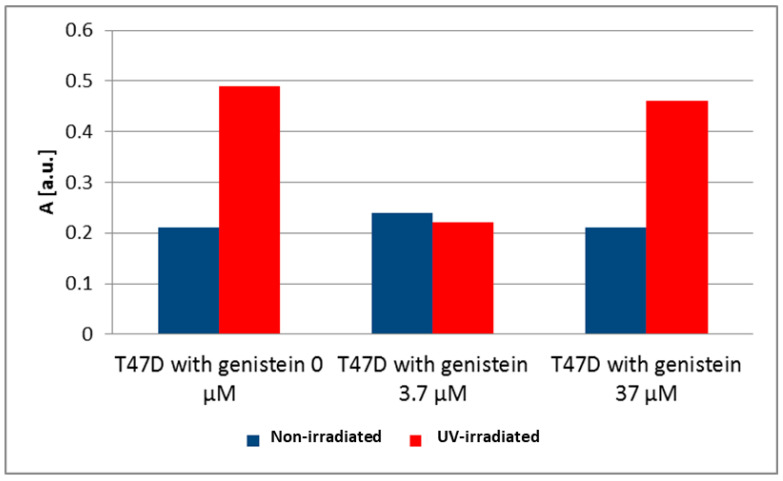
The effect of genistein on the amplitudes (A) [±0.1 a.u.] of the EPR spectra of non-irradiated and UV-irradiated T47D cancer cells. The genistein concentrations were 3.7 μM and 37 μM.

**Figure 6:**
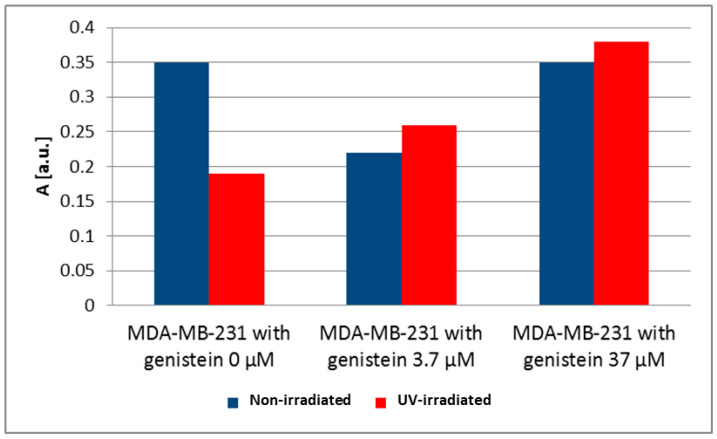
The effect of genistein on the amplitudes (A) [±0.1 a.u.] of the EPR spectra of non-irradiated and UV-irradiated MDA-MB-231 cancer cells. The genistein concentrations were 3.7 μM and 37 μM.

EPR spectra were measured for MCF, T47D and MDA-MB-231 cancer cells. The g-values for all EPR spectra were close to 2, which is characteristic of free radicals. Basal levels of free radicals in breast cancer cells were considered as a control for the cells of each breast cancer cell line (MCF7 0 μM, Figure 4; T47D 0 μM, Figure 5; MDA-MB-231 0 μM, Figure 6). Different amounts of free radicals were present in the different cell types. UV irradiation changed the amount of free radicals in the tested samples. The antioxidant effectiveness of genistein at concentrations of 3.7 µM and 37 µM was related to the level of free radicals in breast cancer cells not treated with genistein (0 μM); UV-irradiated breast cancer cells were compared to non-irradiated breast cancer cells for each breast cancer cell line. In our previous study, the effect of genistein at concentrations of 3.7 µM and 37 µM on the free radicals concentration in both normal dermal (adult NHDF cell line) and keloid fibroblasts (KEL FIB cell line) after ultraviolet irradiation was investigated [33].

The amplitudes (A) of the EPR spectra of non-irradiated and UV-irradiated MCF7, T47D and MDA-MB-231 cancer cells were compared.

The amplitudes (A) and therefore also the amount of free radical in the non-irradiated and UV-irradiated cells increased as follows: T47D cancer cells < MDA-MB-231 cancer cells < MCF7 cancer cells; and UV-irradiated cells increased as follows: MDA-MB-231 cancer cells < MCF7 cancer cells < T47D cancer cells.

One of the key characteristics of cancer cells is increased oxidative stress [34], which can result not only from overproduction of reactive oxygen species, but also from low levels or inactivation of antioxidant mechanisms [12].

Furthermore, the results of Xing et al. [35] showed that more malignant human breast cancer cells are characterized by reduced basal free radical production.

UV irradiation is one of the factors causing the increase of free radicals in cells. UV irradiation caused only a slight decrease in the amount of free radicals in MCF7 cells, resulting in a slight decrease in the amplitude (A) after UV irradiation from 0.68 [a.u.] to 0.46 [a.u.]. A large decrease of the amplitude (A) of the EPR spectra of MDA-MB-231 cancer cells from 0.35 [a.u.] to 0.20 [a.u.] was observed after UV irradiation. The amount of free radicals in UV-irradiated MDA-MB-231 cancer cells was much lower than in non-irradiated cells. The amplitudes (A) and the amount of free radicals in T47D cancer cells increased only slightly after UV irradiation. The amplitude (A) increased from 0.21 [a.u.] to 0.49 [a.u.] for the EPR spectra of these cells.

The decrease in the amount of free radicals may be due to their recombination. In addition, cancer cells have developed mechanisms to protect themselves from oxidative stress and are adapted to redox imbalance, enabling survival [36].

Genistein decreased the amplitudes (A) and the amount of free radicals in both non-irradiated and UV-irradiated MCF7 cells (Figure 4). The amplitudes (A) of the EPR line of MCF7 cells decreased from 0.68 [a.u.] for the control cells to 0.39 [a.u.] (3.7 μM genistein) and to 0.34 [a.u.] (37 μM genistein) for the cells cultured with genistein. As can be seen, only a weak effect of genistein concentrations on free radical levels was observed in non-irradiated cells. The decrease in the amplitudes (A) and the amounts of free radicals in UV-irradiated MCF7 cells caused by genistein were lower than in the non-irradiated MCF7 cells (Figure 4). The amplitude (A) of the EPR line of UV-irradiated control cells was 0.46 [a.u.], and the amplitudes of these cells cultured with genistein were 0.40 [a.u.] (3.7 μM genistein) and 0.39 [a.u.] (37 μM genistein). Similar levels of free radicals were obtained in the UV-irradiated MCF7 cells cultured with genistein at concentrations of 3.7 μM and 37 μM.

Low micromolar concentrations of genistein have shown antioxidant effects, possibly due to its phenolic structure (similar to that of oestrogen) and by upregulating antioxidant gene expression. In addition, genistein reduces basal peroxide levels in MCF7 cells [37]. Genistein has protective effects against cellular damage such as UVB-induced oxidative stress and ionizing radiation-induced damage [38]. Landauer et al. demonstrated a radioprotective property of genistein when administered 24 h prior to ionizing irradiation, but there was no effect when administered 1 h prior to irradiation [39]. The inhibition of growth and induction of cancer cell death (e.g., prostate, breast, lung, pancreatic) achieved by chemotherapeutic drugs (cisplatin, tamoxifen, docetaxel, doxorubicin and gemcitabine) can be enhanced by the use of genistein [40], which modifies the activity of key cell proliferation and survival pathways such as those controlled by Akt, nuclear factor-κB and cyclooxygenase-2 [41].

Genistein did not significantly alter the amplitudes (A) and amounts of free radicals in non-irradiated T47D cancer cells (Figure 5). Genistein at a concentration of 3.7 μM strongly decreased amplitudes (A) (from 0.49 [a.u.] to 0.22 [a.u.]) and free radical levels in UV-irradiated T47D cancer cells (Figure 5). Only the very small decrease in amplitude (A) (from 0.49 [a.u.] to 0.46 [a.u.]) was observed for T47D cancer cells treated with genistein at a concentration of 37 μM. The effect of genistein was then negligible.

Treatment with genistein in the T47D breast cancer cell line resulted in reduced oxidative stress and improved mitochondrial function compared to the MCF7 breast cancer cell line. This may be due to the higher expression of ERβ in T47D cells [42].

Genistein at a concentration of 3.7 μM decreased the amplitude (A) from 0.35 [a.u.] to 0.22 [a.u.]) and the amount of free radicals in non-irradiated MDA-MB-231 cancer cells (Figure 6). Genistein at the higher concentration of 37 μM did not change the amplitude (A) and the amount of free radicals in these non-irradiated cells (Figure 6). The increase of the amplitudes (A) (from 0.19 [a.u.] to 0.26 [a.u.] (3.7 μM genistein) and 0.38 [a.u.] (37 μM genistein) and the amount of free radicals in UV-irradiated MDA-MB-231 cancer cells with increasing genistein concentration was observed (Figure 6).

Results of a study by Kim et al. showed that genistein does not act as an antioxidant, but as a pro-oxidant, in human promyeloid leukaemia HL-60 cells. The pro-oxidant activity of genistein caused a rapid transition of HL-60 cells into the G2/M phase and, thus inhibiting cell proliferation and apoptotic cell death. In addition, the combination of genistein treatment and ionizing radiation showed a synergistic effect on cell death in HL-60 cells, while genistein showed a radioprotective effect in normal lymphocytes [43].

**Figure 7:**
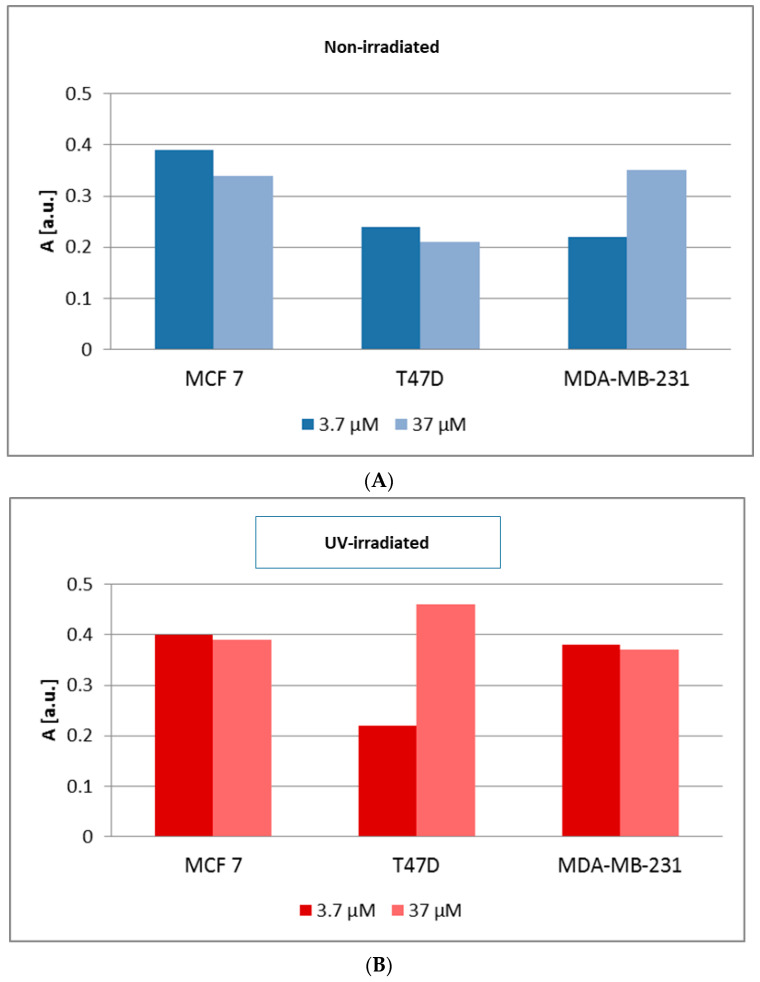
Comparison of the amplitudes (A) [±0.1 a.u.] of the EPR spectra of MCF7 cells, and T47D and MDA-MB-231 cancer cells cultured with genistein at concentrations of 3.7 μM and 37 μM, for non-irradiated (**A**) and UV-irradiated (**B**) cells.

The comparison of the amplitudes (A) [±0.1 a.u.] of the EPR spectra of MCF7 cells and T47D and MDA-MB-231 cancer cells cultured with genistein at concentrations of 3.7 μM and 37 μM, for non-irradiated and UV-irradiated cells, was made in Figure 7. In conclusion, for non-irradiated cells, the strong influence of genistein concentration on the amount of free radicals in MDA-MB-231 cancer cells was obtained. In UV-irradiated MCF7 and MDA-MB-231 cancer cells, genistein concentration had very little effect on free radicals.

Various natural and synthetic chemical compounds can be used to assess antioxidant activity in vitro using a broad spectrum of methods. Most of these methods are based on simple redox reactions between antioxidants and reactive oxygen species, such as ABTS (2,2′-azinobis-(3-ethylbenzothiazoline-6-sulfonic acid), DPPH (2,2′-diphenyl-1-picrylhydrazyl radical), or FRAP (ferric ion reducing antioxidant parameter) tests [44]. These methods for assessing antioxidants do not reflect the true impact of antioxidants on living organisms, as they are used in non-physiological conditions. The assessment of antioxidant properties of natural or chemical compounds is based on the measured activity and gene expression of reduced glutathione, glutathione peroxidase, glutathione reductase, catalase and superoxide dismutase in cells. This indirect assessment was described in a study by Omar Abdulhakeem Almaghrabi, which investigated the effect of quercetin on oxidative stress induced by cisplatin in rat kidney [45]. Electron paramagnetic resonance (EPR) spectroscopy is an effective method for directly measuring free radical production and investigating their intra-cellular concentration [33]. In our study, intra-cellular basal concentration of free radicals in breast cancer cell lines, the antioxidant capacity of genistein in neutralizing free radicals, and the change in the amount of free radicals after UV irradiation directly using electron paramagnetic resonance (EPR) spectroscopy were demonstrated.

One of the main properties of cancer cells is enhanced oxidative stress [34], which can appear not only from reactive oxygen species overproduction, but also from low levels or inactivation of antioxidant mechanisms [36]. Excessive intracellular levels of ROS characterize not only cancer cells but also the tumour microenvironment. ROS in cancer cells additionally stimulate the malignant phenotype by activating continuous proliferation, death avoidance, angiogenesis, invasiveness and metastasis. Presence or absence of receptors for oestrogen, progesterone and human epidermal growth factor receptor 2 HER2 in breast cancer cell lines is also a factor determining different ROS production [46]. Results of a study by Xing et al. [35] revealed that more malignant human breast cancer cells are characterized by reduced production of basal free radicals. One anticancer strategy is based on the redox system of cancer cells. The use of therapies based on generating an increase in the amount of ROS (radiotherapy and photodynamic therapy) is widely used in the treatment of different types of cancers, including breast cancer. Numerous anticancer drugs (i.e., doxorubicin, rituximab, 5-fluorouracil, celecoxib) elevate intracellular ROS level in diverse mechanisms. Increased ROS levels in cancer cells may induce the three types of programmed cell death: the extrinsic/intrinsic pathway of apoptosis, necroptosis and autophagy [47]. Elevated ROS level, a common feature of cancer cells, makes them more susceptible to a further ROS increase compared to normal cells [47,48]. Antioxidants protect the cells against oxidative stress, may act as carcinogenesis attenuators and can be useful in cancer treatment. The potential use of antioxidants to support cancer treatment may arise from their potential chemotherapeutic properties (prooxidant mechanism), their function as an adjuvant, their ability to promote radiosensitization, or their ability to reverse chemoresistance. Antioxidant therapeutic strategies in cancer can be classified as targeting ROS with non-enzymatic antioxidants (such as NRF2 activators or vitamins) or targeting ROS with enzymatic antioxidants, including NAPDH Oxidases (NOX) inhibitors, superoxide dismutase SOD mimics, N-Acetyl cysteine (NAC), and gluthatione (GSH) esters, for cancer treatment. Additionally, it highlights the chemopreventive and chemotherapeutic effects of genistein, which include arresting the cell cycle, inhibiting cell proliferation, inhibiting inflammation and stimulating apoptosis [49].

Possible molecular mechanisms of genistein in preventing breast cancer include decreased response to growth factors due to downregulation of tyrosine kinase activity, G0/G1 arrest by cell cycle transition and G2/M phase arrest via cyclin B. Additionally, genistein may induce apoptosis by downregulating CIP2A mRNA, modulating E2F, inactivating NF-kB, modulating the Bcl-2 Bax ratio, activating caspase-3 and upregulating DNA fragmentation. The anti-proliferative effects of genistein are a result of the upregulation of ERα, decreased ER binding and inhibition of E2-dependent cell growth by Erβ [50].

## 4. Conclusions

Signal transduction associated with oestrogen receptors plays a crucial role in both the pathomechanism and treatment of breast cancer. The literature provides extensive information on the role of exogenous oestrogens in inducing and protecting against carcinogenesis. Naturally occurring oestrogen-like compounds, such as genistein, an isoflavone, can affect the redox potential of cells and may also impact carcinogenesis. The analysis of the effect of genistein on the level of free radicals in an in vitro model, using cell lines with different molecular profiles of signalling pathways associated with carcinogenesis, is of particular interest. The use of the EPR technique in our studies allowed us to obtain interesting data since there are no available data in the literature evaluating the direct measurement of free radicals in breast cancer cell lines. The EPR study revealed that the quantity of free radicals was dependent on the cell type, UV irradiation and genistein interactions. The amount of free radicals increased in non-irradiated and UV-irradiated cells in the following order: T47D cancer cells < MDA-MB-231 cancer cells < MCF7 cancer cells and MDA-MB-231 cancer cells < MCF7 cancer cells < T47D cancer cells. UV irradiation resulted in a slight decrease in the amount of free radicals in MCF7 cells, a significant decrease in the amount of free radicals in MDA-MB-231 cancer cells and a significant increase in the amount of free radicals in T47D cancer cells. Genistein reduced the amount of free radicals in both non-irradiated and UV-irradiated MCF7 cells. At a concentration of 3.7 μM, it significantly decreased the amount of free radicals in UV-irradiated T47D cancer cells. The impact of genistein on the remaining cells was insignificant. At a concentration of 3.7 μM, genistein reduced the amount of free radicals in non-irradiated MDA-MB-231 cancer cells, but this effect was not observed at 37 μM. The amount of free radicals in UV-irradiated MDA-MB-231 cancer cells increased with increasing genistein concentration. EPR studies revealed that genistein affected the free radicals generated in the tested cells.

## Data Availability

The data are available from the corresponding author upon reasonable request.
